# A computational approach to resolve cell level contributions to early glandular epithelial cancer progression

**DOI:** 10.1186/1752-0509-3-122

**Published:** 2009-12-31

**Authors:** Sean HJ Kim, Jayanta Debnath, Keith Mostov, Sunwoo Park, C Anthony Hunt

**Affiliations:** 1 UCSF/UC Berkeley Joint Graduate Group in Bioengineering, University of California, Berkeley, California, 94720, USA; 2 Department of Pathology, University of California, San Francisco, California 94143, USA; 3 Department of Anatomy, University of California, San Francisco, California 94143, USA; 4 Department of Bioengineering and Therapeutic Sciences, University of California, San Francisco, California 94143, USA

## Abstract

**Background:**

Three-dimensional (3D) embedded cell cultures provide an appropriate physiological environment to reconstruct features of early glandular epithelial cancer. Although these are orders of magnitude simpler than tissues, they too are complex systems that have proven challenging to understand. We used agent-based, discrete event simulation modeling methods to build working hypotheses of mechanisms of epithelial 3D culture phenotype and early cancer progression. Starting with an earlier software analogue, we validated an improved in silico epithelial analogue (ISEA) for cardinal features of a normally developed MDCK cyst. A set of axiomatic operating principles defined simulated cell actions. We explored selective disruption of individual simulated cell actions. New framework features enabled recording detailed measures of ISEA cell activities and morphology.

**Results:**

Enabled by a small set of cell operating principles, ISEA cells multiplied and self-organized into cyst-like structures that mimicked those of MDCK cells in a 3D embedded cell culture. Selective disruption of "anoikis" or directional cell division caused the ISEA to develop phenotypic features resembling those of in vitro tumor reconstruction models and cancerous tissues in vivo. Disrupting either process, or both, altered cell activity patterns that resulted in morphologically similar outcomes. Increased disruption led to a prolonged presence of intraluminal cells.

**Conclusions:**

ISEA mechanisms, behaviors, and morphological properties may have biological counterparts. To the extent that in silico-to-in vitro mappings are valid, the results suggest plausible, additional mechanisms of in vitro cancer reconstruction or reversion, and raise potentially significant implications for early cancer diagnosis based on histology. Further ISEA development and use are expected to provide a viable platform to complement in vitro methods for unraveling the mechanistic basis of epithelial morphogenesis and cancer progression.

## Background

Epigenetic deregulation of cell activity is thought to be an important requirement in the preclonal phase of glandular epithelial cancer [1]. What level of deregulation is required before the histology becomes abnormal? Can a mechanism of deregulation be inferred from the abnormal phenotype? To better understand causal linkages between mechanisms and phenotype in an in vitro setting, epithelial cells have been cultured and studied in three-dimensional (3D) gels of extracellular matrix (ECM), such as collagen I or Matrigel^®^. When grown embedded in 3D culture, Madin-Darby canine kidney (MDCK) cells form identically structured acinar organoids enclosing a cell-free, fluid-filled lumen [2]. Proliferation and apoptosis are essential features of the process. When manipulated or exposed to certain factors, the organoids and composing cells exhibit phenotypic features that are associated with pre-cancerous or cancerous tissues in vivo [3]. Such cell culture models are thought to provide an appropriate physiological environment to study glandular epithelial morphogenesis and cancer progression.

From a systems modeling perspective, epithelial cell cultures are abstract, somewhat simplistic models of epithelial cells in vivo. They are constructed wet-lab models: a controllable, careful assemblage of laboratory materials and equipment, in which one component is alive. There is little direct overlap between measured in vitro phenotypic attributes and corresponding attributes of epithelial cells in vivo. Nevertheless, the accumulated literature and the model's continued study attest that scientifically useful mappings exist between model and referent at several levels, from genetic to systemic phenomena [2,4,5]. However, even though the in vitro cultures are orders of magnitude simpler than epithelial cells in a mammalian tissue context, they still are complex systems that have proven challenging to understand.

We suggest that progress can be made in understanding epithelial cell behavior, morphology, and mechanisms, along with the changes that occur during cancer progression by constructing and studying abstract analogues in software, where the system features at all levels can be modeled, fully explored, and understood. The envisioned products are examples of executable biology [6-8], which are useful for systematically exploring hypotheses about referent mechanisms through virtual experimentation. The approach works synergistically with inductive mathematical methods and emerging, hybrid approaches based on "first principle" physical laws [9-11].

In a previous study [12], we presented a cell-mimetic analogue of the envisioned new class, which validated for a small set of targeted MDCK attributes. Its growth characteristics and the types of stable structures formed mimicked those of MDCK cells in cultures. Eleven axiomatic operating principles, and six simulated cell actions, were adequate for validation. However, it failed to consistently produce cystic structures with a round, convex contour, a cardinal feature of normal in vitro phenotype which has not been considered in the earlier study.

Starting with the earlier analogue, we explored several analogue revision strategies to achieve the expanded attribute set. For the purposes of this study, we focused on cell event (death and division) patterns and multicellular morphology. Because of the networked nature of axiom use, some changes intended to have one effect also had other, unintended, often abiotic consequences. One of our guidelines was to keep revisions parsimonious. We sought one new analogue having as few new axioms as possible to achieve morphological validation against the shape requirement, in addition to the original set of target attributes. For one, validation was achieved by addition of only one new cell action coupled with replacement of one axiom. Use of the new action enabled the improved in silico epithelial analogue (ISEA) to form stable cystic structures with smooth, convex margins similar to those observed normally in 3D epithelial cell culture.

We reasoned that if a mapping exists between ISEA's coarse-grained operating principles and the more complex epithelial cell counterparts, then selective disruption of ISEA's operation should exhibit cancer-like characteristics of in vitro epithelial cancer reconstruction models [13], examples of which are provided in the Appendix. We designed and implemented methods to selectively deregulate, at a controlled level of severity, simulated cell operation at the axiom level. We focused on two processes known to be critical for normal ISEA growth and stabilization. One process ended with an ISEA version of anoikis, a specific form of cell death due to extracellular matrix detachment. The other involved directed placement of a daughter cell, the ISEA's version of oriented cell division. A grading measure was developed and used to quantify changes in morphology.

Dysregulation of either "anoikis" or directed daughter cell placement, or both, led to manifest changes in ISEA phenotype that were reminiscent of dysplastic growth associated with in vitro cancer reconstruction and early glandular epithelial cancer progression in vivo. Consequently, we undertook a detailed analysis of the deregulations and their consequences. Varying the level of dysregulation led to morphologies that could be classified into groups using automated grading. Dysregulation of ISEA's anoikis process had a greater effect on overall morphology. Simultaneous dysregulation of the two axioms had a nonadditive effect. Importantly, dysregulation of either process resulted in similar morphological outcomes, which could not be differentiated reliably without additional information on growth dynamics, with potentially significant implications for early cancer diagnosis based on histology. The results also provided an early lead on possible additional mechanisms to reconstruct, or revert, cancer-like phenotypes in experimental and therapeutic contexts. Future rounds of development of these in silico methods may lead to a viable platform for unraveling the operational bases of glandular epithelial morphogenesis and early cancer progression.

## Methods

The ISEA design is based on the methods and principles of agent-based modeling [14] and discrete event simulation [15]. The ISEA and its predecessors [12] can be categorized broadly as cell-based or cell-centered models, which encompass cellular automata, cellular Potts models, and various types of agent-based or individual-based models as reviewed in [8,16,17]. Cellular automata and their relationship to ISEA are discussed in the Appendix.

Detailed descriptions of ISEA components and the experimentation framework are provided in [12,18]. An abridged description follows. To clearly distinguish ISEA components and processes from their in vitro counterparts, hereafter we use small caps when referring the former. As detailed in [18], ISEA is a standalone system that comprises CULTURE analogue and system-level components for semi-automated experimentation and analysis. System-level components include EXPERIMENT MANAGER, OBSERVER, and CULTURE graphical user interface (GUI). EXPERIMENT MANAGER, the top-level agent, provides experiment protocol functions and specifications. OBSERVER agent is responsible for recording CULTURE measurements. CULTURE GUI enables visualization and user interaction during simulation.

A CULTURE is an agent that maps abstractly to the MDCK cell culture within one well of a multi-well culture plate. It maintains a two-dimensional (2D) hexagonal grid, which represents an arbitrary cross-section through a 3D MDCK cell culture. The grid has toroidal topologies. Discrete objects with eponymous names represent the essential cell culture components: CELLS, MATRIX, and FREE SPACE. MATRIX and FREE SPACE are passive objects that map to units of extracellular matrix (ECM) and matrix-free material. A MATRIX object maps to a cell-sized volume of ECM. For simplicity, MATRIX represents any media containing sufficient ECM to which MDCK cells can attach. For the traits targeted, no distinction was needed for differential physical ECM characteristics, such as stiffness, density, and viscoelasticity. A FREE SPACE object maps to a similarly sized volume of material that is essentially free of cells and matrix elements. FREE SPACE also maps to luminal space and non-matrix material in pockets enclosed by cells. The latter are called LUMINAL SPACE when distinction from FREE SPACE is useful.

**Figure 1:**
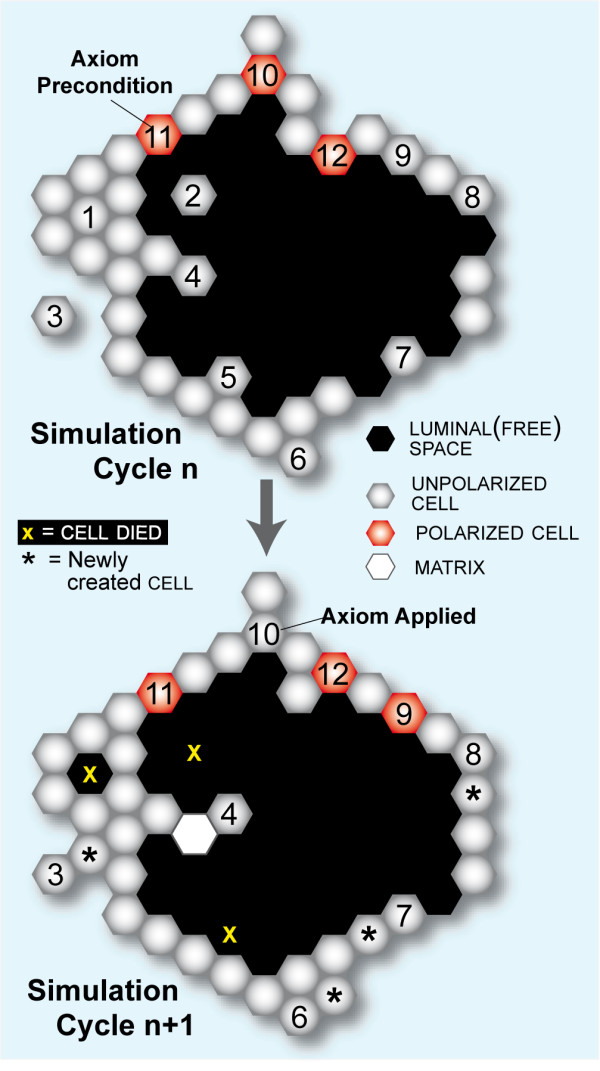
**The twelve ISEA axiomatic operating principles**. Table 1 is a listing and explanation of the operating principles. The 2D space and all objects within are hexagonally discretized. Simulation time advances in steps corresponding to simulation cycles. During a simulation cycle, every CELL, in a pseudo-random order, decides what action to take based on its internal state (POLARIZED or UNPOLARIZED) and the composition of its adjacent neighborhood. A set of axioms determines what action is taken for each possible neighborhood configurations. Objects represented: POLARIZED CELL (red), UNPOLARIZED CELL (gray), MATRIX (white), and LUMINAL SPACE (black). At the top, selected decision-making CELLS at the start of simulation cycle *n *are numbered to indicate each of the twelve axiomatic preconditions being satisfied (they are listed in Table 1). For purpose of this illustration, the unnumbered CELLS are inactive. At the bottom, the system at the start of simulation cycle *n *+ 1 shows the consequences of applying all twelve axioms.

CELLS are quasi-autonomous agents. They use the axiomatic operating principles and decision logic (Table 1; Fig. 1) to interact with components in their local environment. Every CELL has the same step function in which an assessment of its environment is made and a call is made for an appropriate action. The step function is scheduled each simulation cycle. A set of CELL axioms, discussed below, determines CELL action. A CELL selects just one axiom and completes its corresponding action during each simulation cycle.

**Table 1:** ISEA CELL axioms

Axiom	Precondition*	Action	References
1	CELLS only	DIE and leave behind a LUMINAL SPACE	[3,5,51,52]

2	LUMINAL SPACE only	DIE and leave behind a LUMINAL SPACE	[3,5,51,52]

3	MATRIX only	DIVIDE; the daughter CELL replaces a randomly selected MATRIX	[3,22,53]

4	1 CELL and LUMINAL SPACES; no MATRIX	Produce and deposit MATRIX between self and the adjoining CELL	[21,54]

5	≥2 CELLS and LUMINAL SPACE; no MATRIX	DIE and leave behind a LUMINAL SPACE	[19,20,51]

6	≥1 CELL and MATRIX; no LUMINAL SPACE	DIVIDE; the daughter CELL replaces MATRIX that maximizes its number of CELL neighbors	[23,53,54]

7	MATRIX and ≥2 LUMINAL SPACES; no CELLS	DIVIDE; the daughter CELL replaces LUMINAL SPACE that adjoins MATRIX	[2,23]

8	CELLS, MATRIX, and ≥2 adjacent LUMINAL SPACES	DIVIDE; the daughter CELL replaces LUMINAL SPACE that adjoins MATRIX and LUMINAL SPACE	[2,23]

9	2 CELLS, MATRIX, and LUMINAL SPACE; POLARIZING condition^†^	POLARIZE	[2,5]

10	DEPOLARIZING condition^‡^	DEPOLARIZE	[2,5]

11	All other configurations	Do nothing	[2]

12	POLARIZING condition; 1 MATRIX	Move and replace the neighboring MATRIX; leave behind a LUMINAL SPACE	[2,5]

* The precondition for each axiomatic operating principle (Fig. 1) takes into consideration the six objects adjacent to the decision-making CELL. ^† ^Two CELL neighbors separate MATRIX on one side from LUMINAL SPACE on the other side. ^‡ ^A POLARIZED CELL has noncontiguous MATRIX neighbors.

### CELL axiomatic operating principles

An agent must have rules and protocols for interacting with adjacent components. The operating premise is that the same is true for cells in culture; what can be described as rules and protocols are emergent properties of the cell's expressed genetics and ongoing biochemistry. Rules can take any form. We elected to have each rule take the form of an axiom, which specifies a precondition and corresponding action. Preconditions correspond to a CELL'S neighborhood configurations. For action options, we specified what we determined to be a minimal set to achieve validation: replace an adjacent non-CELL object with a CELL copy, DIE (vanish) and leave behind a LUMINAL SPACE, create MATRIX, destroy an adjacent non-CELL object and move to that location leaving behind a LUMINAL SPACE, POLARIZE, DEPOLARIZE, and do nothing. For any precondition, only one action was executed.

Detailed descriptions of supporting biological evidence and assumptions made for ISEA CELL axioms are provided in [12,18]. Briefly, CELL DEATH axioms (Axioms 1, 2, and 5) were based on a general biological principle that cells, such as epithelial cells, undergo a process of cell death within some interval after detaching from ECM [19,20]. That behavior is observed in MDCK cell cultures [3,5]. Axiom 4, which dictates MATRIX deposition between two adjacent CELLS, was specified based on observations that some matrix is produced de novo between two adhering MDCK cells in suspension culture [21]. A CELL DIVISION axiom, Axiom 3, follows from experimental observations that, when embedded in matrix, single MDCK cells proliferate [3,22]. Other CELL DIVISION axioms, Axioms 6, 7, and 8, follow from a similar, general principle that epithelial cells proliferate when they adhere to ECM and tend do so in arrangements that maximize intercellular contact [2,23]. CELL POLARIZATION axioms, Axioms 9 and 10, reflect in vitro observations on MDCK cell polarity [2,5]. Axiom 11 applied when the CELL achieved mandates that map to the three-surfaces principle articulated in [2]. An UNPOLARIZED state indicates that the CELL'S three surfaces mandate has not been achieved.

The earlier axioms developed by Grant et al. [12] enabled the analogue to validate for a set of basic MDCK cell culture attributes. Unlike its referent, the model frequently produced highly irregular, abiotic structures. We revised the axioms to enable ISEA to consistently develop CYSTS having roundish, convex shapes, a cardinal feature of a normal in vitro epithelial cyst. Note that a regular hexagon in a hexagonally discretized space maps to a circle in continuous space. Fig. 1 describes and shows use of all 12 ISEA axioms. Axioms 1-10 were carried forward from [12]. ISEA variants that were more elaborate also enabled ISEA variants to achieve the targeted attributes, but they were rejected because we strove to adhere to the guideline of parsimony.

We considered and explored adding and using limited details about the axis of POLARIZATION. A CELL, following one of its action options, acquiring CELL neighbor or prior to creating a copy, for example, could assign itself a vectorial axis of POLARIZATION. Thereafter, an axiomatic precondition could include reference to the direction of POLARIZATION combined with neighborhood information. However, other analogues that were explored, including ISEA, achieved the targeted attributes without that added detail. Adhering to the parsimony guideline, we excluded POLARIZATION details because they were not needed to achieve the attributes targeted, but they can be added easily when the need arises. Axiom 8 requires a CELL to be aware of the positions of its neighboring objects relative to each other and itself. A higher resolution (more fine-grained) mechanism that would be exchangeable for Axiom 8 could include having and using an axis of POLARIZATION.

### Operational disruption of CELL axioms

Following implementation (and validation) of the revised axiomatic operating principles, our next task was to add a mechanism to disrupt the operation of individual CELL axioms selectively. We added a set of parameters, one per axiom, which controlled the probability of the decision-making CELL electing to follow the axiom when its precondition applies. The parameter values ranged from 0 to 1 inclusively. A parameter value = 1 corresponded to 100% adherence to the axiom. Setting the parameter to zero completely blocked execution of the prescribed action and, if specified, dictated an alternate action. At its decision point, each CELL drew a pseudo-random number (PRN) from the standard uniform distribution. The prescribed action was followed only when the PRN was ≤ the probability threshold set for the corresponding parameter.

Following exploratory simulations that considered many options, we specified alternative actions that mapped to plausible in vitro cell actions in a deregulated state. Axioms 1, 2, and 5 governed CELL DEATH; a reasonable alternative was to resist DEATH and remain ALIVE (i.e., do nothing). Axiom 3 dictated random placement of a CELL copy; its alternate action was to do nothing and thus prevent REPLICATION. We also assigned the alternate action of 'do nothing' to Axiom 4 (MATRIX production). Several dysregulated action options were available to Axiom 6 (ORIENTED CELL DIVISION). One was to do nothing, effectively suppressing CELL DIVISION. Another was undirected CELL DIVISION, placing the CELL copy in a random direction without regard for the number of CELL neighbors. We used the latter because adequate, supportive biological information was available. Axiom 7, which dictated CELL DIVISION, had available the same alternative action options. Axiom 8 (CELL DIVISION or POLARIZATION) had many plausible options. One was preventing CELL DIVISION; another, as above, was to allow the CELL to place a copy of itself in any available location. Yet, another option was to enable POLARIZATION. The preconditions prescribing CELL DIVISION or POLARIZATION also could be swapped. The remaining axioms, Axioms 9-12, posed a similar problem of having many plausible action options. Because no experimental information was available to narrow the options, we elected to defer investigation of those axioms until more information becomes available.

### Simulation experiments

CULTURE width and height were set to 100. For EMBEDDED CELL CULTURE simulation, a single CELL was placed at the center of the CULTURE grid filled with MATRIX. One CELL width mapped to 10 μm. Simulation time advanced discretely. Ordering of CELL events within the same simulation cycle was pseudo-random. Each simulation experiment comprised 100 Monte Carlo (MC) runs. Each MC run was executed for 50 simulation cycles. One simulation cycle mapped to 12 h in vitro. A new CULTURE was created for each repetition.

### Specification and use of morphology index

The morphology index, *M*, weighs three basic features of MULTICELL morphology: local EXTRACELLULAR arrangement, *E*, structural discontinuity, *D*, and a structure's overall shape, *S*. For each CELL, the algorithm computes a numerical score based on its neighborhood arrangement. An ideal arrangement corresponds to the Axiom 12 precondition. Higher scores are assigned to neighborhood configurations that deviate from that ideal. The collective EXTRACELLULAR arrangement score, *E*, is the mean of individual CELL scores. A CLUSTER is structurally continuous so long as it remains one connected body of CELLS and FREE (or LUMINAL) SPACE. When structural continuity is broken, two or more CLUSTERS are formed. The structural discontinuity algorithm computes the number of disconnected bodies; that number translates to *D*. The shape profile algorithm takes into consideration a structure's overall 2D shape in hexagonal space and computes a score, *S*, which increases as shape becomes irregular or deviates from the ideal shape, a regular hexagon (a regular hexagon in hexagonal space maps to a circle in continuous space). The value of the morphology index becomes *M *= *E *+ *D *+ *S*. The maximum values of *E*, *D*, and *S *have been set to 3, 2, and 1 respectively, reflecting their assigned relative weights. The final morphology index value ranges from 1 (an ideal CYST) to 6 (disorganized). Lower scores are assigned to configurations that are more organized and roundish with a single LUMEN. The measure was implemented and calibrated for ISEA simulations, and sufficed for this study's purposes. However, we will need counterpart metrics for in vitro and in vivo microscopic image analysis should we undertake direct, quantitative comparison between ISEA and wet-lab morphologies. A detailed description of the measure and algorithms are provided in additional file 1: Supplementary Material.

### Tools used for analogue implementation and execution

The model framework was implemented using MASON Version 11. MASON is a discrete-event, multi-agent simulation library coded in Java [24]. Batch simulation experiments were performed on a small-scale Beowulf cluster system. Computer codes and project files are available at http://biosystems.ucsf.edu/research_epimorph.html

## Results

### ISEA was validated against normal 3D embedded cell culture traits

We implemented a common framework and components, some derived from the earlier analogue [12]. Having a new general framework was needed in part to reduce unnecessary, cross-model redundancies between different candidate CELLS during analogue refinement, and facilitate an iterative model refinement process capable of automated cross-model validation. As done in [12], we validated the revised ISEA for all four growth conditions: monolayer, overlay, suspension, and embedded cultures. Simulation results for monolayer and suspension CULTURES were identical to those in [12]. Results for overlay cultures were closer in appearance to in vitro observations (not shown). Marked differences were observed for the EMBEDDED CULTURE condition. That CULTURE condition is the focus hereafter.

**Figure 2:**
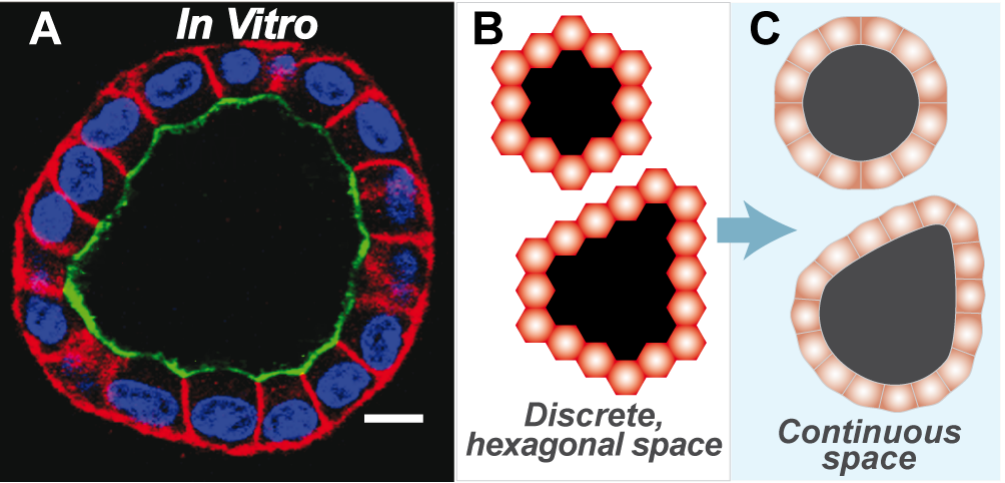
**MDCK and simulated cysts**. (A) MDCK cells grown in 3D extracellular matrix form lumen-enclosing cystic structures surrounded by a layer of polarized cells. Cells composing cysts maintain three surfaces: apical (red), basal and lateral (green). Note the roundish contour typical of MDCK cysts. For growth and staining details, see [22]. Bar: ~10 μm. (B) Representative, stable ISEA CYSTS. CELLS in EMBEDDED condition produced stable, cystic structures enclosing LUMINAL SPACE; all CELLS were POLARIZED (red). CYSTS had convex shapes. (C) This illustration shows that convex polygonal CYSTS in discretized 2D hexagonal space map to a roundish structures in continuous 2D space. Because such a mapping provides no added scientific or mechanistic information, subsequent ISEA structures are shown as they appeared at simulation's end in 2D hexagonal space.

At the start of an EMBEDDED CULTURE simulation, a single CELL was placed in CULTURE space, surrounded by only MATRIX. As a simulation progressed, the CELL underwent repeated rounds of CELL REPLICATION, followed by the formation of LUMINAL SPACE and an increase in CELL number and CYST diameter. The central LUMINAL SPACE grew as CELLS in the inner region DIED or moved out. The growth dynamics and final morphology were similar to those observed for MDCK cells (Fig. 2A). The EMBEDDED CULTURE always formed stable CYSTS bordered by POLARIZED CELLS (Fig. 2B), and ISEA consistently produced CYSTS with a roundish, convex shape with smooth margins. During an occasional simulation, because of their changing, local environment, one or more CYST surface CELLS failed to POLARIZE or DIE before the simulation ended. Such events prevented the local rounding out process that a POLARIZED CELL can undertake, preventing a few structures from stabilizing within 50 simulation cycles.

### New capabilities were added to simulate epigenetic deregulation of cell processes

Following validation, we undertook experiments that may simulate inducement and progression of in vitro cell phenotypes that mimic early, preclonal stages of carcinogenesis. The progression involves alterations in tissue organization and morphology but low genetic changes [1]. The experiments were motivated by questions such as these: what happens when normal cell operation and its regulation become faulty? To what extent can individual cell activities be disrupted separately and together, and still maintain an apparently normal morphology? What visible changes accompany relaxation of tight control of a cell-level process? Can the operational cause of the changes be predicted from histological morphology? To obtain answers for ISEA, we conducted experiments in which the probability (*p*) of proper axiom operation was varied from 1 to 0. When proper operation was not followed, alternate, dysregulated actions were used. From an operational standpoint, having *p *= 0 represents a permanent change in the CELL'S operating principles throughout the simulation. A nonzero value below 1.0 specifies a reversible change in the CELL'S operation, or erratic operation. It can be viewed either as the probability of the CELL behaving properly at any point in time, or the approximate percentage of time that the CELL acts normal (i.e., strictly adheres to the axiomatic operating principles illustrated in Fig. 1) during simulation. Individual CELLS decide to act normal (or not) each simulation cycle, independent of one another. As a result, CELLS can exhibit different behaviors (normal vs dysregulated) during a simulation cycle. With *p *< 1, a CELL can switch between the two behaviors multiple times during a simulation. It is a stochastic process having the Markov property: future changes in the CELL'S behavior occur probabilistically, independent of its history.

We focused on Axioms 5 and 6. Axioms 2, 3, 4, and 7 were not essential to normal (i.e., validated) CYST formation in EMBEDDED CULTURE (but they were needed for other simulated culture conditions), and were used infrequently. Consequently, they were excluded from this investigation. As described in Methods, selecting an alternate action for disrupted Axioms 8-11 is not straightforward. We elected not to pursue disruption of those axioms until further insight from wet-lab studies becomes available to narrow options. Disrupting Axiom 1 was straightforward but the outcomes (not shown) offered no significant insight: CELL CLUSTERS either developed normally into CYSTS (*p *> 0) or grew unchecked as a homogenous mass (*p *= 0). We expected that outcome because Axiom 1 is required for LUMINAL SPACE creation but becomes nonessential as soon as the nascent LUMINAL SPACE is formed. Axioms 5 and 6 were essential to CYST formation in EMBEDDED CULTURE. Axiom 5 dictates ANOIKIS (in silico counterpart to anoikis, a form of cell death), and is the most frequently used CELL DEATH axiom during CULTURE growth. Axiom 6 dictates oriented CELL DIVISION; the action requires making a CELL copy and placing it selectively. That process accounts for most of the CELL DIVISION events in the simulations. Their biological counterparts are centrally implicated in epithelial morphogenesis and carcinogenesis.

To aid investigation, we developed and used a morphology index to automatically quantify ISEA structure morphology. The algorithm analyzed and scored features of MULTICELL structures. The measure weighed three characteristics: local EXTRACELLULAR arrangement, structural continuity, and the overall shape. The index values ranged from 1 to 6. Higher values indicated a more disorganized state. Lower scores were assigned when the overall shape was convex, and all CELLS were POLARIZED. The measure was calibrated for ISEAs, but could be generalized for other model types. The index represents the simplest metric that we found to be sufficiently discriminative for this study's purposes. We are exploring alternative metrics that may expedite simulation analysis and interpretation. However, it is unlikely that a single, simple metric will suffice for analyzing multiple, distinguishable morphological features, each of which requires different measurements to quantify and characterize. It is more likely that the complexity of the metric will parallel the complexity of the multi-feature attributes being analyzed. A further discussion of the morphology index is provided in additional file 1: Supplementary Material.

### Reduced ANOIKIS caused LUMEN filling

For comparison, selected examples of in vitro structures formed following dysregulation of normal cell processes are provided in the Appendix.

**Figure 3:**
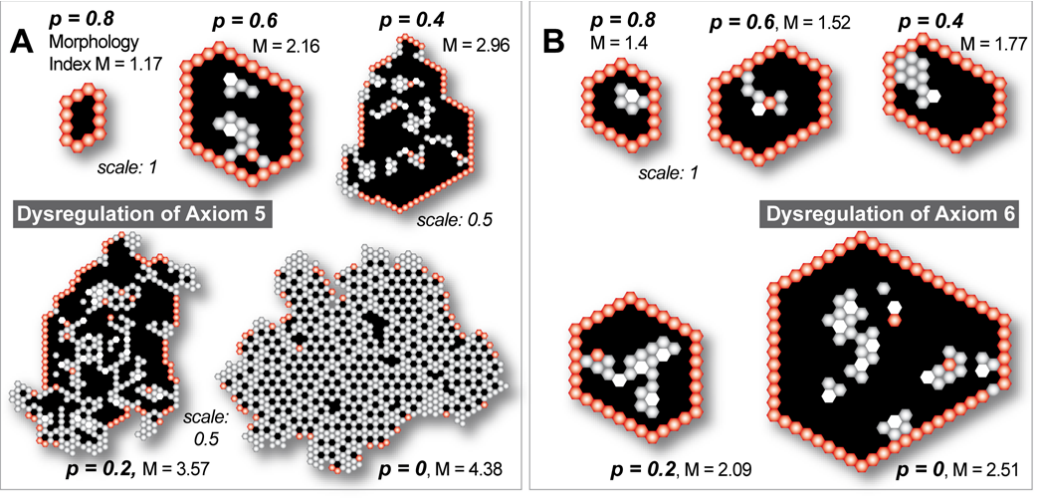
**Dysregulation of Axiom 5 or 6 has a disruptive effect on ISEA CULTURE morphology in a severity-dependent manner**. Axiom 5 dictates ANOIKIS (which maps to a form of cell death) when the CELL in its neighborhood has at least two CELLS and LUMINAL SPACE but no MATRIX. In simulations dysregulating Axiom 5, CELLS evaded ANOIKIS (by doing nothing) with a parameter-controlled probability, *p*, when Axiom 5's precondition was met. Axiom 6 dictates oriented CELL DIVISION when the CELL has at least one CELL and MATRIX but no FREE SPACE. When Axiom 6 was dysregulated, CELLS carried out disoriented CELL DIVISION: the CELL copy replaced a randomly selected MATRIX neighbor without regard for CELL neighbor number. Shown are CULTURE images and corresponding morphology index values after 50 simulation cycles of growth. One simulation cycle maps to 12 h in vitro. Each object is represented as a hexagon: POLARIZED CELL (red), UNPOLARIZED CELL (gray), MATRIX (white), and LUMINAL SPACE (black). One CELL width maps to 10 μm. (A) Axiom 5 dysregulation caused progressively disorganized CULTURE formations. (B) Axiom 6 dysregulation showed a similarly severity-dependent effect. The changes were less prominent but nevertheless clearly aberrant in both analogues.

When cultured within 3D ECM, normal epithelial cells typically proliferate and organize into hollow spheroids, a process that recapitulates certain structural features of a glandular epithelium, such as the presence of a central, hollow lumen [2]. In MDCK and some mammary cell cultures, apoptosis contributes centrally to lumen formation: cells in the inner region of the developing structure undergo anoikis upon loss of direct matrix contact [25]. Blocking anoikis in vitro has been shown to cause lumen filling, which resembles a characteristic of most glandular epithelial cancers [3]. In 3D cultures of mammary epithelial cell line MCF-10A, focal adhesion kinase-mediated inhibition of apoptosis by inhibition of metalloproteinase 1 (TIMP1) has been shown to induce lumen filling and disrupt normal acinar development [26]. Also, expression of oncoproteins with anti-apoptotic activities, including receptor tyrosine kinase ErbB2, colony-stimulating factor 1 receptor, SRC, and IGF1R, has been shown to elicit abnormal, filled phenotypes that resemble human ductal carcinoma in vivo [13]. If ISEA's operating principles have cell culture counterparts, then simulation results should exhibit (predict) LUMEN filling when ANOIKIS is compromised. We simulated the condition by disrupting Axiom 5 use, which allowed CELLS lacking MATRIX contact (e.g., enclosed in the CYST LUMINAL SPACE) to evade DEATH for one or more simulation cycles. With nonzero *p *< 1, anoikis was disrupted transiently, not permanently for any one CELL. In simulations with Axiom 5's *p *= 0, CELLS were essentially immune from DEATH on MATRIX detachment except when completely enclosed by other CELLS (Axiom 1). ANOIKIS dysregulation caused aberrant growth morphology (Fig. 3A), and changed CELL DEATH and DIVISION activity patterns.

**Figure 4:**
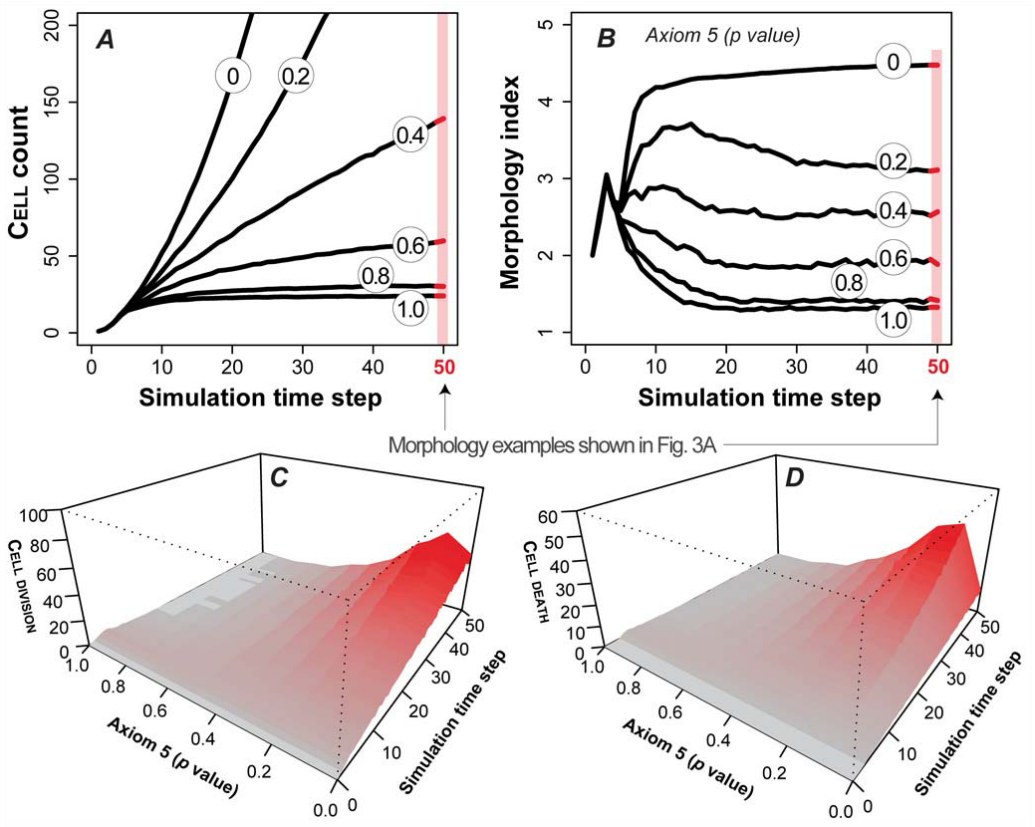
**Dysregulation of Axiom 5 (ANOIKIS) and its effect on ISEA CULTURE growth and morphology**. Axiom 5 dictates CELL DEATH when a CELL has in its neighborhood at least two CELLS and LUMINAL SPACE but no MATRIX. With a parameter-controlled probability, *p*, CELLS evaded ANOIKIS (by doing nothing) when Axiom 5's precondition was met. Doing so caused distinct changes in growth and structural characteristics of the EMBEDDED CULTURE. (A) CELL CULTURE growth rate increased monotonically with the severity of dysregulation. CULTURE growths at six levels of dysregulation are shown. (B) Disrupting operation of Axiom 5 resulted in the formation of progressively aberrant MULTICELL structures, as indicated by the numeric scale. Higher values indicate a more disorganized morphology. Dysregulation had no observable effect on CULTURE morphology in the early stages (~5 simulation cycles) of growth. One simulation cycle maps to 12 h in vitro. The effect became progressively evident as simulation time advanced. (C-D) Axiom 5 dysregulation altered CELL DIVISION and DEATH event patterns. The changes became more evident at later times. In both simulations, the effect on CELL DEATH and DIVISION was monotonic, except when *p *= 0. The mean occurrence of CELL DIVISION and DEATH fell when *p *= 0 (vs *p *= 0.8). The data are mean values of 100 Monte Carlo runs.

For *p *< 1, mean ISEA CULTURE growth rates increased nonlinearly with increasing dysregulation. As evident in Fig. 4A, CULTURE growth became unchecked as CELLS evaded ANOIKIS more frequently. Growth became nonlinear at the lowest *p *values. CULTURE morphology also exhibited changes (Fig. 4B), becoming more aberrant with increased dysregulation. However, dysregulation had only a limited impact on morphology index during the early proliferative stage of ≤ 5 simulation cycles. Thereafter, values either decreased or increased further, depending on the degree of dysregulation. Increased dysregulation always led to a higher mean index value at simulation's end. Images recorded after 50 simulation cycles showed more irregularities and larger structures following increased dysregulation (Fig. 3A).

The structures exhibited distinctive morphologies depending on dysregulation level. For instance, 'maximal' dysregulation (*p *= 0) resulted in an aggressively expanding CELL mass with minimal POLARIZATION, which failed to develop a central LUMINAL SPACE. The neighborhoods of most CELLS consisted of CELLS and LUMINAL SPACE. On a gross level, the morphological features resembled those associated with in vitro transformation by inhibition of anoikis or apoptosis [13]. Less dysregulation resulted in structures in which portions exhibited somewhat normal attributes, including the formation of a central LUMINAL SPACE and increased POLARIZATION along the margins. However, CELLS remained in the LUMINAL SPACE, and most were UNPOLARIZED.

Dysregulation also affected CELL activity patterns. In particular, the number of CELL DEATH and CELL DIVISION occurrences rose steeply over the growth period under severe dysregulation (*p *≤ 0.4), as illustrated in Fig. 4C-D. The increase was not as dramatic, and tended to remain relatively steady with less dysregulation. A drop in CELL DIVISIONS was observed when ANOIKIS was blocked (*p *= 0) relative to strong dysregulation (*p *= 0.1). The result was unexpected given the virtually unchecked CULTURE growth measured under that condition. However, even though growth was unchecked, the elimination of ANOIKIS reduced the relative frequency of CELL DIVISION opportunities compared to extensive but incomplete inhibition of Axiom 5 use. Analysis of individual axiom use and their relative frequencies (additional file 1: Supplementary Material) showed that the relative frequency of CELL DIVISION (as a consequence of Axiom 6 use) exceeded the use of all CELL DEATH axioms by a factor of > 2 when *p *= 0. There was a several-fold increase in Axiom 1 activity, but it failed to compensate for the absence of Axiom 5 use; Axiom 2 use showed no observable increase. In partially dysregulated conditions (*p *= 0.4-0.8), Axioms 1 and 2 showed little activity after the first few simulation cycles. CELL DEATH and DIVISION caused by Axioms 5 and 8 occurred more frequently as simulation progressed, indicative of active PROLIFERATION and DEATH of CELLS in contact with LUMINAL SPACE. Both exhibited similar use frequencies and changes over time.

### Dysregulation of oriented CELL division disrupted CYST formation

Oriented cell division is central to multicellular morphogenesis [27-29]. Its disruption is implicated in cancer progression [30]. The cell division axis orientation determines the position of the daughter cells, their contents and hence their fate. It has been shown that both matrix contact and cell adhesions play important roles in determining the orientation of the division axis in vitro [31,32]. In MDCK cell cultures, disruption of cell polarity by ablating the mammalian ortholog of PALS1, a gene involved in epithelial polarity and division orientation in Drosophila, results in incomplete, multiple lumen formations [33]. Also, in Drosophila *aurA*, *mud*, and *polo *mutants, improper cell division axis orientation results in abnormal accumulation of dividing cells and tumor development [30]. What impact would deregulation of oriented cell division have on 3D epithelial cell culture phenotype? If any, could it recapitulate features of early cancer progression? Cell axis and orientation are below the current ISEA resolution. Nevertheless, to the degree that the low granularity mappings between ISEA and in vitro systems are acceptable, a dysregulated form of Axiom 6, which accounts for most of the CELL DIVISION events that occur during CULTURE growth, can be used to explore plausible answers. To achieve that aim, we dysregulated Axiom 6 by allowing the DIVIDING CELL to place its daughter CELL in a randomly selected MATRIX location (vs one that maximizes CELL contact). We anticipated that, if Axiom 6's operation maps abstractly to a form of oriented cell division in vitro, then the resulting CULTURE phenotype would provide insight into the expected role of oriented cell division in the development, or disruption, of epithelial architecture in vitro.

**Figure 5:**
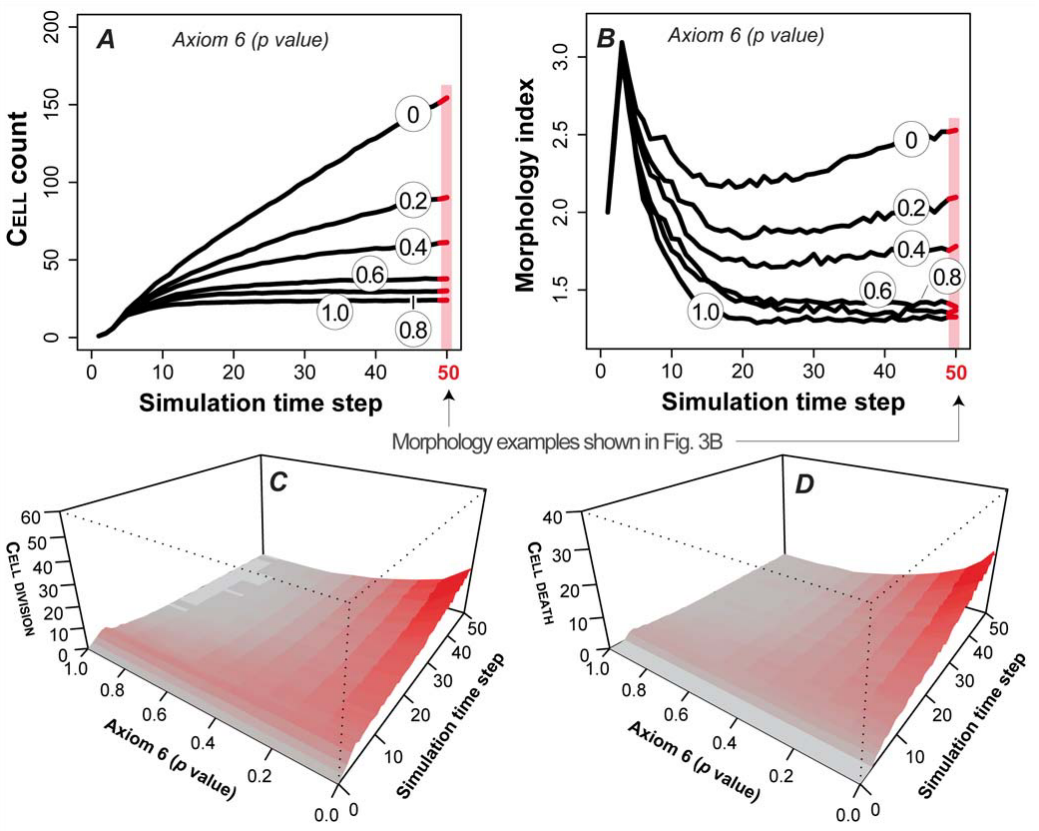
**Axiom 6 (oriented CELL DIVISION) dysregulation and its effect on ISEA CULTURE growth and morphology**. Axiom 6 dictates CELL DIVISION when a CELL has at least one CELL and MATRIX but no FREE SPACE in its neighborhood. The CELL copy is placed at an adjacent MATRIX position that maximizes its number of CELL neighbors. With a parameter-controlled probability, *p*, CELLS followed an alternate, dysregulated action (disoriented CELL DIVISION) when the Axiom 6 precondition was met. The CELL copy replaced a randomly selected MATRIX neighbor without regard for CELL neighbor number. Doing so caused changes in growth and structural characteristics of the EMBEDDED CULTURE. (A) CELL CULTURE growth rate increased monotonically with the severity of dysregulation. (B) Shown are changes in growth morphology. Similar to Axiom 5 dysregulation, this analogue showed no observable effects during the early growth stage but obvious differences over time. (C-D) Axiom 6 dysregulation altered CELL DIVISION and DEATH event patterns. Near the maximally dysregulated state (*p *= 0), the system exhibited a proportionately larger increase in CELL DEATH events at later times. The data are mean values of 100 Monte Carlo runs.

We ran simulations with Axiom 6's *p *ranging from 0 to 1 and recorded changes in CULTURE growth, morphology, and CELL activity patterns. Some results are shown in Figs. 3B and 5. CULTURE growth rate increased monotonically with dysregulation (Fig. 5A). The changes were less dramatic than those observed when Axiom 5 was dysregulated. With maximal dysregulation, mean CELL population after 50 simulation cycles reached 150 CELLS, compared with 900 CELLS following Axiom 5 dysregulation. CULTURE morphology also exhibited changes (Fig. 5B). When severely dysregulated (*p *≤ 0.4), the developing structures exhibited increasing morphological irregularities. The increase correlated with the presence of UNPOLARIZED CELLS inside the LUMINAL SPACE (Fig. 3B). Using CULTURE GUI, we visualized CULTURE growth and observed CELLS undergoing continual, active cycles of PROLIFERATION and DEATH inside the LUMINAL SPACE.

CELL DEATH and DIVISION activities (Fig. 5 C-D) continued to register as simulations progressed when highly dysregulated. In fact, the mean number of CELL DIVISIONS and CELL DEATH occurrences increased over time. CELL DEATH events were offset by an approximately equal number of cell divisions. Their apparent dynamic balance resembled how a hollow structure is maintained by the increased apoptosis of cells inside the lumen when proliferation is increased in mammary epithelial cell culture [3].

Axiom 6 use accounted for most CELL DIVISION events during early growth (additional file 1: Supplementary Material), but in a more severely dysregulated state, Axiom 8 (another driver of CELL DIVISION) was used more frequently as simulations progressed. The increase in CELL DIVISION was offset by a similar increase in Axiom 5 (ANOIKIS) use frequency. Decreased use of Axiom 12 (do nothing) provided further evidence for the continual, dynamic CELL turnover occurring inside the LUMINAL SPACE during growth. That is because current ISEA axiom use assumes that nutrient availability is the same in LUMEN as in the EXTRACELLULAR CULTURE. If that is not the case, then it is straightforward to make axiom use frequencies nutrient dependent.

### Combined dysregulation of ANOIKIS and oriented CELL DIVISION caused phenotypic changes reminiscent of early phase cancer progression

**Figure 6:**
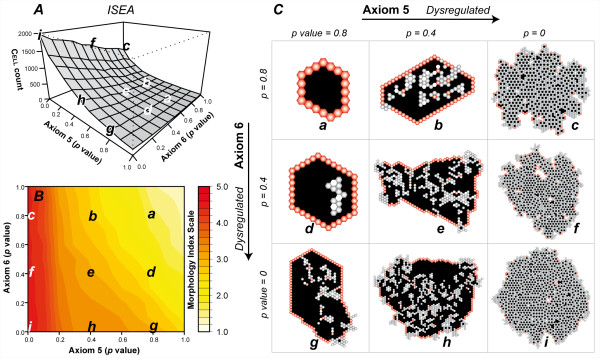
**Simultaneous dysregulation of Axioms 5 and 6 and its effect on ISEA CULTURE growth and morphology**. Axioms 5 and 6 dictate ANOIKIS (a form of CELL DEATH) and oriented CELL DIVISION; both are essential to normal CYST growth in EMBEDDED CULTURE. With a parameter-controlled probability, *p*, for each of the two axioms, CELLS followed an alternate, dysregulated action. For Axiom 5, the alternate action was to evade ANOIKIS (i.e., do nothing). For Axiom 6, it was disoriented CELL DIVISION; the CELL copy replaces a randomly selected matrix neighbor. The lower case letters (a-i) in (A) and (B) correspond to the morphologies in (C). (A) ISEA CELL population, (B) morphology index values, and (C) simulation images after 50 simulation cycles of growth. One simulation cycle maps to 12 h in vitro. Each object is represented as a hexagon: POLARIZED CELL (red), UNPOLARIZED CELL (gray), MATRIX (white), and LUMINAL SPACE (black). One CELL width maps to 10 μm. The CELL count and morphology measurements are mean values of 100 Monte Carlo runs.

We also explored conditions in which Axioms 5 and 6 were dysregulated simultaneously. The experiments may map to experimental manipulation of protein complexes such as activated receptor tyrosine kinases in epithelial cell culture, which can deregulate both cell division and cell death processes [34]. We varied the two axioms' *p *independent of each other and conducted 100 Monte Carlo simulation experiments for each condition. The dysregulated ISEA, illustrated in Fig. 6, exhibited nonlinear growth changes. Change was most striking for the maximally dysregulated condition (*p *= 0), which resulted in a mean cell population of ~2000 CELLS vs ~900 CELLS when only Axiom 5 was dysregulated, or 150 CELLS from deregulating only Axiom 6. Changes at other tested levels were also nonlinear (Fig. 6A). As measured by CELL count and morphology index (Fig. 6A, B), Axiom 5 dysregulation contributed more to observed phenotypic changes. When Axiom 5 was maximally dysregulated, ISEA produced structures with morphology index values > 4, regardless of Axiom 6's *p *(Fig. 6B). Simulation images (Fig. 6C) recorded after 50 simulation cycles showed differentiable morphologies that roughly coincided with different gradations observed in Fig. 6B. The altered ISEA morphologies mapped to characteristics of the in vitro cancer reconstruction model and early cancer progression in vivo [1,13,35].

In results from the above experiments, we observed similar morphologies regardless of which axiom was dysregulated. No new features emerged from simultaneous dysregulation of Axioms 5 and 6. For example, note the CULTURE images in Figs. 3A (*p *= 0.6), 3B (*p *= 0.2), and 6C (Axiom 5's *p *= 0.8 and Axiom 6's *p *= 0.4). The similar features included formation of a central LUMINAL SPACE, which is fully enclosed by a monolayer of POLARIZED CELLS, and the presence of mostly UNPOLARIZED CELLS in the inner region. The similarities were reflected in the morphology index measurements. Consequently, we could not infer from CULTURE morphology alone which axiom (Axiom 5, 6, or both) had been dysregulated. However, making such a determination is straightforward given CELL axiom use patterns. In time, gene or protein expression patterns of individual cells may emerge as the wet-lab counterpart to axiom use patterns.

## Discussion

Studies of epithelial cell cultures are providing knowledge about how individual cell activities are mediated by intrinsic and environmental factors to create the diverse phenotypes of normal epithelial morphogenesis and epithelial cancers. There is a need for additional methods to facilitate achieving a deeper, integrated understanding of the growing body of experimental observations. Past efforts have demonstrated how combined experimental and computational approaches contribute to that process [36,37]. Our goal is to broaden and strengthen that effort by developing software analogues that are useful 1) as instantiated, working hypotheses of epithelial morphogenesis and tumorigenic phenotype in vitro, and 2) as an extensible, interactive resource of available biological knowledge about the mechanisms implicated in those processes. Progress described herein represents an early step towards achieving those goals.

We revised and extended the axiomatic operating principles of an earlier model [12] to those shown in Fig. 1. The revised ISEA consistently produced roundish, convex CYSTS with smooth margins, a cardinal feature of normal in vitro MDCK phenotype. We enabled mechanistic tracing during simulations of all processes essential for normal ISEA development. Two critical axioms were targeted for dysregulation: Axiom 5, which controlled ANOIKIS, and Axiom 6 that dictated an abstract form of oriented cell division. The causal chains of events responsible for ISEA phenotype were explored in detail following dysregulation, a process which is infeasible using current state-of-the-art in vitro methods.

Dysregulated ISEA morphology exhibited features reminiscent of those associated with in vitro cancer reconstruction models and early cancer progression in vivo (see selected in vitro images in the Appendix). By increasing dysregulation of the two axioms, we altered ISEA morphology progressively to mimic features of epigenetic change that accompany early precursor lesions like atypical ductal hyperplasia [1]. ISEAs using dysregulated ANOIKIS (Axiom 5) developed MULTICELLULAR structures having ill-formed LUMINAL SPACES containing disorganized nests of CELLS. With increased dysregulation, LUMINAL CELLS sometimes broke out through the enclosing monolayer to PROLIFERATE into the surrounding MATRIX, as illustrated in Fig. 6C. Although such behavior has not been observed in studies of apoptosis inhibition in 3D culture, the activation of certain growth factor receptors able to promote luminal space survival, such as ErbB2, do exhibit similar expansive phenotypes in 3D [3,34]. If a mapping does exist between those ISEA behaviors and phenomena of epithelial systems, it suggests that epigenetic changes may be capable of inducing invasive behaviors in otherwise apparently normal cells in vitro or in vivo [1,38]. The phenomena merits further in silico exploration.

Similar, but less dramatic changes were observed when we dysregulated oriented CELL DIVISION (Axiom 6). Simultaneous dysregulation of the two axioms produced nonadditive effects but no new morphological features emerged: the structures were virtually indistinguishable from those obtained by dysregulating only Axiom 5 or 6. Consequently, without a priori dysregulation knowledge, one would be unable to reliably deduce the operational cause of a change in CULTURE phenotype based solely on morphology images. A similar conclusion has been reached based on in vitro findings that phenotypic changes such as lumen filling in 3D cultures can be induced by deregulation of different molecular mechanisms [13]. To the extent that the in silico-to-in vitro and in vitro-to-in vivo mappings are valid, the results support the idea that morphologically similar dysplasia can have different causes, and that may have implications for early diagnosis of cancer based on morphology alone, as very aggressive, early stage cancers may appear morphologically similar to potentially less aggressive, abnormal, non-cancerous growths.

Dysregulation of either axiom enabled some CELLS to survive in the LUMINAL SPACE. That ISEA behavior maps to in vitro observations [13,35]. How the latter occurs has not been determined. How it occurs within ISEA may provide insight. A subset of INTRALUMINAL CELLS established MATRIX contact by producing MATRIX de novo (via Axiom 4 use). So doing enabled them and some other CELLS to survive in aggregates inside the LUMINAL SPACE, where they underwent cycles of PROLIFERATION and DEATH. Blocking the CELLS' ability to produce matrix (Axiom 4) reduced INTRALUMINAL CELL survival dramatically, and facilitated clearing of residual INTRALUMINAL CELLS during LUMINAL development (data not shown). In vitro, similar phenomena have been observed in MCF-10A epithelial cell cultures: cells accumulated inside cyst lumens when an anti-apoptotic protein, Bcl-2 was overexpressed [3]. However, unlike in ISEA simulation, the cells eventually died and disappeared. Mechanisms underlying the latter process are unknown. Interestingly, some evidence suggests that Bcl-2 activates matrix metalloproteinase (MMP), which degrades ECM surrounding cells [39]. Do the above INTRALUMINAL CELL survival observations have an in vitro counterpart, or are these ISEA behaviors outside phenotype overlap? If there is an in vitro counterpart, then intraluminal epithelial cells in 3D embedded culture may evade apoptosis and further insure their survival by secreting matrix de novo for anchorage. In such a scenario, MMP activation could have an opposing effect by degrading the cell-secreted matrix, rendering the cells vulnerable to anchorage-dependent anoikis.

Dysregulation of Axiom 6 demonstrated the importance of proper DIVISION direction during CULTURE growth. Evidence supports a mapping to in vitro counterparts. Similar structures form when cell polarity is disrupted in MDCK cell cultures by ablating the mammalian ortholog of PALS1, a gene involved in epithelial polarity in Drosophila [33]. Similar to ISEA behaviors, the structures contain multiple intraluminal cell clusters and resemble certain patterns observed in breast ductal carcinomas in situ and prostate hyperplasia [35]. Because cell polarity is critical to cell division orientation, one could speculate that a disruption in oriented cell division by PALS1 ablation may have contributed to the observed phenomenon. We also note that several groups have discovered that Ric-8 protein plays a key part in the positioning of the division axis in Drosophila morphogenesis [40,41]. It is not yet known if Ric-8 plays a similar role in oriented mammalian cell division in cultures. Nevertheless, ISEA behaviors indicate that compromising one or more of the mechanisms managing oriented cell division can contribute to features that mimic early stage, cancer-like structures in 3D cultures.

The ISEA methods used to mimic attributes of cancer reconstruction can be compared to those used to model tumor growth in vitro and in vivo. Recent models [42-46] have represented cancerous cells as permanently transformed cell line. We explored incremental dysregulation of specific ISEA mechanisms. Galle et al. [47] used a similar, creative, individual cell-based approach to simulate and study epithelial cell monolayer growth. They used selective "knockouts" of cell level growth regulation and control mechanisms to investigate how those different mechanisms collectively acted together to influence population morphology. More recently, Rejniak and Anderson [48,49] introduced single cell-based, immersed boundary simulation models of epithelial acini development in vitro, and applied the models to investigate different conditions of growth that contribute to normal and abnormal acinar development. Other studies have used single cell-based cellular Potts models and extensions to simulate various aspects of development including embryonic cell patterning and tumor invasion [16,17].

Finally, CELL axioms are high level, low-resolution placeholders for more detailed representations of the actual complex mechanisms driving epithelial cell behavior. Use of axioms precludes explicit representations of the abundant, detailed subcellular information that is available. However, starting with the current more abstract set of axioms provided the simplest method and approach for building a useful, working model, positing principles of operation, and testing hypotheses as discussed above. On the other hand, a key advantage of the approach built into ISEA and its framework are their adaptability for inclusion of additional attributes and details through an iterative model refinement process [8]. The current analogue and its components, including CELL axioms, can be further developed to reflect new biological information (e.g., cell positioning mechanisms). We can elaborate ISEA to include higher granularity components and mechanisms that map to subcellular details such as cell lifecycle pathways and intercellular signaling networks when validation against an expanded set of targeted attributes requires doing so. From an engineering perspective, doing so is straightforward and can be accomplished by swapping the current component (e.g., CELL) for a more detailed composite agent as described in additional file 1: Supplementary Material. Replacement could also occur at the intra-component level, for example by replacing CELL axioms with more detailed logic based on interacting components. A challenging task will be to insure cross-model validation between the different analogue variants, and to develop appropriate automated validation measures.

## Conclusions

The approach described herein enabled instantiating a working hypothesis of how individual epithelial cell actions may give rise to cyst organization in vitro, and when disrupted selectively, to structures having tumor-like characteristics. Modest dysregulation of one of two key ISEA operating principles was sufficient to cause manifest changes in its original morphology. The results support the position that epigenetic deregulation of a cell's principles of operation is sufficient to cause emergence of attributes of early stage cancers. We anticipate future rounds of ISEA refinement and validation will provide an additional, viable experimental approach to dissect the operational basis of glandular epithelial morphogenesis and cancer progression.

## Authors' contributions

SK and CH conceived the idea. SK designed and performed the experiments. SP participated in the design and implementation. SK, JD, KM, and CH analyzed the experiment results. SK and CH wrote the paper with input from coauthors. All authors read and approved the final manuscript.

## Appendix

### Relationships between ISEA and cellular automata

It can be useful to relate agent-based models to cellular automata. A CA consists of a regular grid of "cells" which transition through a series of states in discrete time steps. The "cells" are immobile. A "cell" transitions its state based on the states of neighboring "cells". Transitions are synchronous, meaning that all "cells" are updated each time step. A global software executive controls state transitions and time evolution. A fundamental attribute of CA is the realization of non-local, complex behaviors arising from the operation of local rules [50]. A CA can be thought of as a simple type of object-oriented program (OOP), where objects confined to specific locations map to a CA's "cells" and the transition rules are the objects' methods. The only differences being that 1) the objects in an OOP can determine with which other objects they interact, 2) their interactions are not necessarily synchronous, and 3) any object may have more than a single state transition rule. In essence, such an OOP can be viewed as being a more heterogeneous and dynamic type of CA.

An agent-based system (ABS) adds considerable heterogeneity over and above that of an OOP. Whereas an OOP is not necessarily synchronous, the control of when an object interacts with another object and which objects interact is still handled by a global executive. Objects are reactive slaves to this global executive, even in a parallelized OOP. Within an ABS, on the other hand, some of the executive's capabilities and responsibilities, including some or all of the scheduling of actions, are distributed--delegated--to agents. An agent can be quasi-autonomous. It senses and is part of its environment, which may or may not be discretized in the form of a grid. It pursues an agenda within a larger script. An agent can choose dynamically with which other agents or objects to interact, when to engage other agents or objects, and which of various actions to take. It can also begin engaging in new actions without being told to do so or how to do so by a global executive. Likewise, it can decide to stop engaging in a given interaction. In fact, an agent can initiate or end the execution of any of its logic, internal or interactive. Given those attributes, "agent" can be defined technically as an object within an OOP with the ability to schedule its own actions. In models such as an ISEA, an agent, like an actor, plays a role, participates in a process, or acts on behalf of something else. Importantly, an agent is identifiable by an observer as a cause of an effect. Some of an agent's attributes and actions may be designed to represent biological counterparts; others will deal with issues of software execution.

It can be important to distinguish an ABS from an agent-oriented system. In the former, all the capabilities described exist in the software itself. In the latter, the actual software may not have all the capabilities of an ABS, but when the system is used, it is useful to think of the simulation as being composed of agents. In that sense, a CA may be agent-oriented but not agent-based. However, an ABS is sufficiently far removed from a CA so that the analogy only has pedagogical value.

### In vitro morphology observations

**Figure 7:**
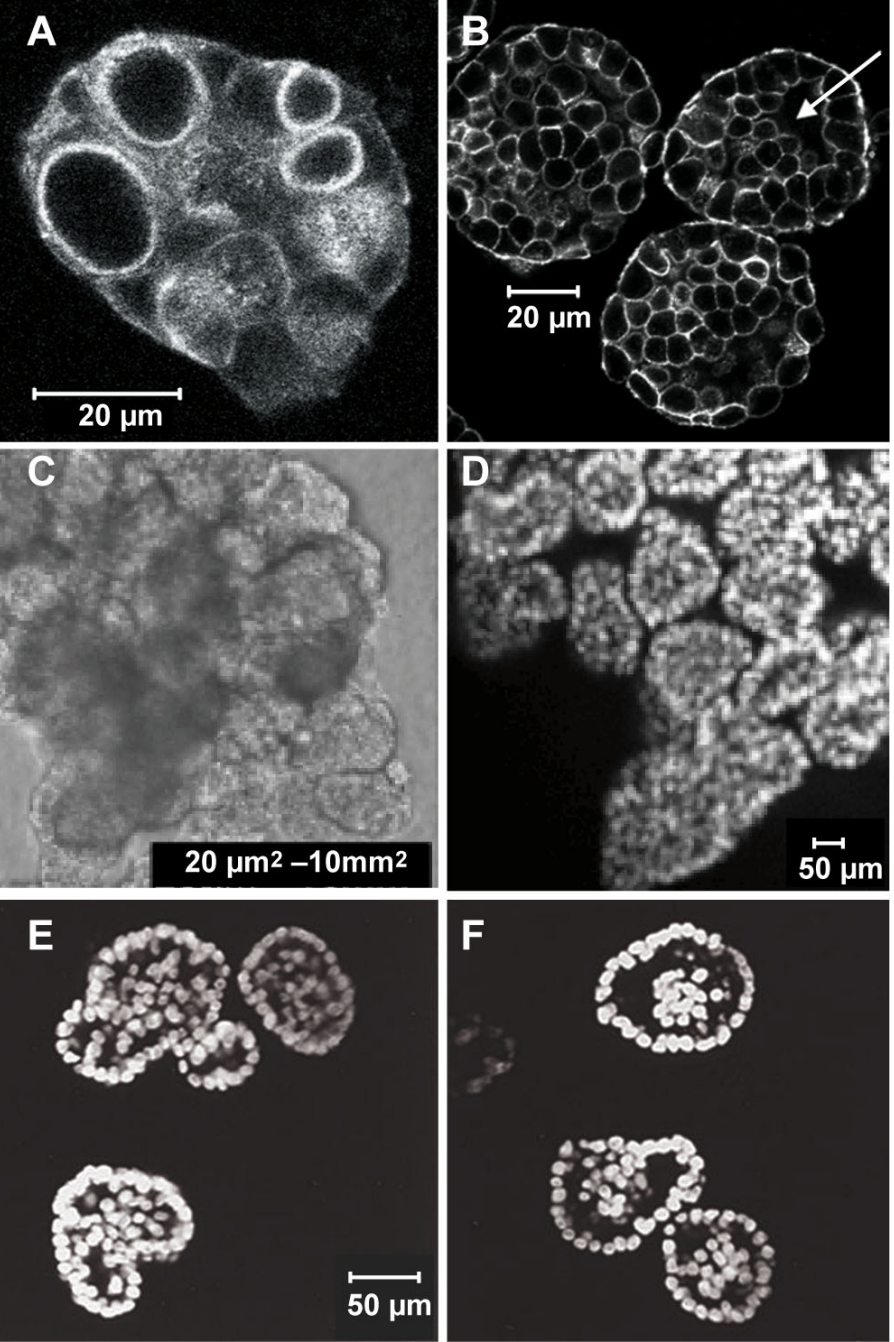
**Microscopic images of in vitro epithelial cysts exhibiting characteristics of early-stage epithelial glandular tumor**. (A-B) Loss of PALS1 expression results in the disruption of MDCK cell polarity and impaired development of cyst lumen [33]. PALS1 is involved in the establishment of cell polarity. Cysts composed of PALS1-ablated cells exhibited multiple small lumens (A) or developed a larger but incomplete lumen (arrow in B). (C-D) Overexpression of ErbB2 receptor leads to the formation of multi-acinar structures with filled lumens in 3D Matrigel [34]. (C) The structures consisted of multiple acinar-like units with filled lumens. The size range of at least 200 structures is shown. (D) Optical section of a single structure along the z-axis. (E-F) Inhibition of luminal apoptosis in proliferating structures results in lumen filling [3]. MCF-10A cells were infected with retroviruses encoding expression of proliferative oncoproteins--cyclin D1 or human papilloma virus (HPV) 16 E7--and anti-apoptotic Bcl family proteins (Bcl-2 or Bcl-X_L_). (E) Acinar structures formed by cells expressing HPV 16 E7 and Bcl-2. (F) Structures formed by cells expressing cyclin D1 and Bcl-X_L_. Panels A-B were reproduced with permission from [33]^© ^The American Society for Cell Biology. Panels C-D were reproduced with permission from [34]^© ^Nature Publishing Group. Panels E-F were reproduced with permission from [3]^© ^Nature Publishing Group.

A number of molecules contribute to the establishment of cell polarity and orientation in mammalian epithelial cells. Among these are PALS1 (Proteins Associated with Lin Seven 1) and PATJ (PALS1-Associated Tight Junction protein). They form macromolecular complexes at tight junctions. Straight et al. [33] ablated expression of PALS1 in MDCK cells in a cyst formation assay, and that led to defects in polarity determination and the failure of cysts to properly form a lumen. Microscopic images taken at day 10 are shown in Fig. 7A-B. Cell masses contained either no lumen or several smaller lumens (Fig. 7A). Occasionally a larger, but incomplete lumen was observed (arrow, Fig. 7B). In ISEA simulations, similar structures were observed when Axiom 6 was dysregulated. ISEA CELLS developed multiple, relatively intact LUMENS at different, early time points, which disappeared or merged into a larger LUMEN by simulation cycle 50 (data not shown). Some were observed at or around simulation cycle 20, which maps to the time (day 10) that the in vitro images were captured. These observed in silico features were mostly transient. Time-lapse images at longer time points will be needed to confirm (or dispute) the in silico observations.

Although MCF-10A and MDCK are different in several ways, when grown under identical 3D culture conditions, their structure formation processes exhibit many similarities, including formation of cysts having similar characteristics. We posit that for those conditions, the current ISEA is also an acceptable analogue of MCF-10A cyst formation. Overexpression of ErbB2 oncoprotein receptors and their epidermal growth factor (EGF) ligands is implicated in epithelial glandular cancer progression. To examine the effects of activating ErbB receptors in a 3D in vitro context, Muthuswamy et al. [34] activated selected ErbB receptors in preformed acinar structures composed of MCF-10A mammary epithelial cells. To create stable cell lines expressing chimeric ErbB2 receptors, cells were infected with retroviruses encoding the chimeras that could be activated by synthetic dimerizing ligands without interfering with endogenous receptors and vice versa. When cultured in 3D Matrigel, the cells proliferated and organized into polarized, lumen-enclosing cysts. Upon activation of the chimera, these cysts developed structures consisting of multiple acinar-like units with filled lumen (Fig. 7C-D). The units within the multi-acinar structures were connected to each other at the base. These altered structures exhibited characteristics of early-stage epithelial tumors, including a high level of proliferation, loss of polarized organization, filling of the lumen, and retention of the basement membrane. Most structures were at least 10 times larger than normal acini (~100 μm); some were 100 times larger (Fig. 7C). They are similar to the ISEA morphology resulting from dysregulation of both Axioms 5 and 6, which could map to the in vitro condition where apoptosis, proliferation, and cell polarity are disrupted by ErbB2 expression. As discussed in Results, severely dysregulated CELLS developed large, expanding CELL masses with numerous, incomplete LUMENS. Like their in vitro counterparts, the ISEA structures were poorly POLARIZED. Without proper POLARIZATION, CELLS at the periphery continued to DIVIDE and expand outward. By simulation cycle 50, some structures became at least 100-fold larger than stable, 'normal' CYSTS. An example is shown in Fig. 6C.

Debnath et al. [3] used MCF-10A cell cultures to analyze the role of apoptosis in the formation and maintenance of luminal space during the in vitro morphogenesis of oncogene-expressing mammary epithelial acini. They made two interventions: one to increase proliferation and another to inhibit apoptosis. Proliferation was increased via the ectopic expression of cyclin D1 or the inactivation of the retinoblastoma protein tumor suppressor pathway by the E7 oncoprotein from human papilloma virus (HPV) 16. Apoptosis was inhibited by infecting cells with retroviruses encoding for exogenous expression of anti-apoptotic Bcl family proteins. Fig. 7E-F shows typical structures formed when cells expressed both the proliferative and anti-apoptotic proteins. Those cells produced acinar structures with partially or completely filled lumen. Similar, filled acinar structures were observed in another study, in which TIMP1, a potent cell survival oncoprotein, was used to inhibit both intrinsic and extrinsic apoptosis [26]. Axiom 5 dysregulation simulated a similar in vitro condition where anoikis, a specific form of cell death associated with extrinsic apoptosis pathway, is disrupted. The dysregulation resulted in structures (Fig. 3A) that exhibited morphological characteristics similar to those shown in Fig. 7E-F. Most structures had CELLS inside the CYST LUMINAL SPACE.

## Supplementary Material

Additional file 1 **Supplementary Material**. Provided are detailed descriptions of ISEA morphology index, ISEA CELL axiom use patterns following Axiom 5 or 6 dysregulation, and a diagram illustrating model refinement and cross-model validation. Click here for file
