# Supplementary material for: A computational approach to resolve cell level contributions to early glandular epithelial cancer progression
Source: BMC Syst Biol. 2009 Dec 31;3:122. doi: 10.1186/1752-0509-3-122 (PMC2814811; doi:10.1186/1752-0509-3-122)
Supplement: Additional file 1 — Supplementary Material. Provided are detailed descriptions of ISEA morphology index, ISEA CELL axiom use patterns following Axiom 5 or 6 dysregulation, and a diagram illustrating model refinement and cross-model validation. [file 1752-0509-3-122-S1.PDF]

Electronic Supplementary Material
for
A Computational Approach to Resolve Cell Level Mechanisms of Early Glandular Epithelial Cancer Progression

Sean HJ Kim, Jayanta Debnath, Keith Mostov, Sunwoo Park, and C Anthony Hunt

Text S1: ISEA morphology index

ISEA morphology index ($M = E + D + S$) was created in part to facilitate and automate morphology analysis, an essential aspect of our study, which otherwise would have required manual, visual screening of simulation images following changes to analogue implementation or parameterization. Conceptual parallels can be drawn between the morphology index and automated methods of classifying tumor histology. For automated tumor grading, various methods of image processing along with correlation-based techniques have been devised to grade histological images. Included are hybrid segmentation methods [1] that quantify morphological features into numerical scores. Similar to those methods, the morphology index provides a numeric scale to classify multi-aspect features of ISEA morphology at the conclusion of each simulation.

A CELL'S E value is determined as follows. When there is one neighboring object type, $E = 2$. When there are two neighboring object types, $E = 2$ if the two object types are separated; $E = 3$ if they are mixed. When there are three neighboring object types, $E = 1$ if matrix and free space are separated by two nonadjacent CELLS; $E = 2$ if MATRIX and FREE SPACE are separated and there are either four CELL neighbors or there are three CELL neighbors adjacent to each other; $E = 3$ if MATRIX and FREE SPACE are adjacent; $E = 3$ if MATRIX and FREE SPACE are separated and there are three nonadjacent CELLS.

The structural discontinuity value, D, is computed by counting disconnected groups of CELLS and FREE SPACE objects. We adapted basic graph methods [2] to identify the isolated groups. From the CELL and FREE SPACE positioning information, separate, unordered adjacency lists of CELLS and FREE SPACE objects are created. Every entry is a set of two neighboring CELLS or FREE SPACE objects. Next, depth-first searches are made on the lists to compute the number of strongly connected groups of CELLS or FREE SPACE. Every strongly connected group represents an unbroken body of CELLS or FREE SPACE. For example, the search will identify two strongly connected groups of CELLS when applied to a CYST with a clump of CELLS isolated within its LUMEN, or one strongly connected group if the CYST is attached to at least one INTRALUMINAL CELL. Because a CYST'S LUMINAL SPACE is continuous, the method will compute one strongly connected group of FREE SPACE in either example. Discontinuity scores for CELLS and FREE SPACE objects are computed separately and summed to render the final discontinuity score. The individual scores are computed from the number, n, of strongly connected groups of CELLS or FREE SPACE as follows:

$$D(n) = \begin{cases} 0 & \text{if } n \leq n_{min} \\ D_{max} & \text{if } n \geq n_{max} \\ D_{max} \times \frac{(n - n_{min})}{(n_{max} - n_{min})} & \text{otherwise} \end{cases} \quad (1)$$

D_{max} is the maximum possible score, n_{min} is the ideal number of strongly connected groups, and n_{max} is the minimum number of strongly connected groups that automatically yields the maximal score D_{max}. For this study, $D_{max} = n_{min} = 1$. The total number of CELLS or FREE SPACE objects is multiplied by 0.15 to compute n_{max} that represents the threshold fraction of strongly connected composing objects or groups of objects, above which automatically yields D_{max}. The value was calibrated to 0.15 to provide adequate sensitivity for ISEA simulations. We assume that MULTICELL structures grown in a culture are maximally disjointed if they have more than n_{max} disconnected bodies of CELLS or FREE SPACE objects.

For each CYST c, the shape algorithm computes and assigns a value, S. The ideal shape in MDCK cultures is spherical with circular cross-sections, which corresponds to a regular hexagon in ISEA's EMBEDDED CULTURE simulation. The algorithm uses a basic shape analysis method to measure the deviation of ISEA structures from the ideal. The algorithm computes S based on the ratio of the area (A_c) enclosed by c to the hexagonal area ($\hat{A}_c$) enclosed by an ellipse circumscribing c as follows:

$$S(c) = min\left(S_{max}, \left|1 - \frac{A_c}{\widehat{A}_c}\right|\right), \text{where } \widehat{A}_c = 2\sqrt{3}ab \quad (2)$$

$S_{max} = 1.0$ is the maximum score; a and b are semimajor (one half the major) and semiminor (one half the minor) axes of the enclosing ellipse. The major axis of the ellipse corresponds to either the length or height of c, whichever is greater; the lesser becomes the minor axis. Lower $S(c)$ values are preferred. Simulated structures with nonconvex or irregular shapes are assigned high scores, while convex contours generally have scores closer to zero. Examples of nonconvex forms include a sickle-shaped CYST with a U-shaped contour. This area-based method suffices as a simple but effective means of quantitatively assessing cyst shape irregularity. Note that shape analysis is a well-studied domain of mathematics and computer science [3], from which methods that are more elaborate can be drawn when needed.

The ISEA morphology index was intended to quantify multi-aspect structural features of EMBEDDED CULTURE growth. For the purposes of our study, we placed more weight on an individual CELLS' neighborhood arrangement, in line with the cell-level perspective of the three-surfaces principle [4]. Less weight was placed on global features. Consequently, the metric highlights features of CELL-level organization that may not be obvious initially upon visual inspection. The relative weights can be changed to enhance the global perspective, for example, overall shape. In its current form, the index is more sensitive to changes in the local CELL environment, which correlate directly with CELL events, hence the dynamic phenotype, and allows for better mechanistic tracking. In its current form, the index is dependent on hexagonal discretization, although its underlying method is invariant to model design and discretization. Similar methods could be developed to automatically grade morphological features of the 3D epithelial cell

culture. Having such information will facilitate concretizing ISEA-to-in vitro mappings and thus automated ISEA refinement as new observations accumulate. Subcellular details, like nuclear morphometry, which has been shown to correlate with tissue phenotype [5] are below the current level of resolution. Likewise, pleometric features (e.g., cell size and shape) that are important indicators of cell malignancy are below the current level of resolution. ISEA's design anticipates inclusion of such features through iterative refinement.

References

1. Petushi S, Garcia FU, Haber MM, Katsinis C, Tozeren A: **Large-scale computations on histology images reveal grade-differentiating parameters for breast cancer.** *BMC Med Imaging* 2006, **6:**14.
2. Gross J, Yellen J: *Graph theory and its applications*. Florida: CRC Press; 2005.
3. Costa LD, Cesar RM: *Shape analysis and classification: theory and practice*. Florida: CRC Press; 2000.
4. O'Brien LE, Zegers MM, Mostov KE: **Building epithelial architecture: insights from three-dimensional culture models.** *Nat Rev Mol Cell Biol* 2002, **3:**531-537.
5. Hoque A, Lippman SM, Boiko IV, Atkinson EN, Sneige N, Sahin A, Weber DM, Risin S, Lagios MD, Schwarting R, Colburn WJ, Dhingra K, Follen M, Kelloff GJ, Boone CW, Hittelman WN: **Quantitative nuclear morphometry by image analysis for prediction of recurrence of ductal carcinoma in situ of the breast.** *Cancer Epidemiol Biomarkers Prev* 2001, **10:**249-259.

Supplementary Figures

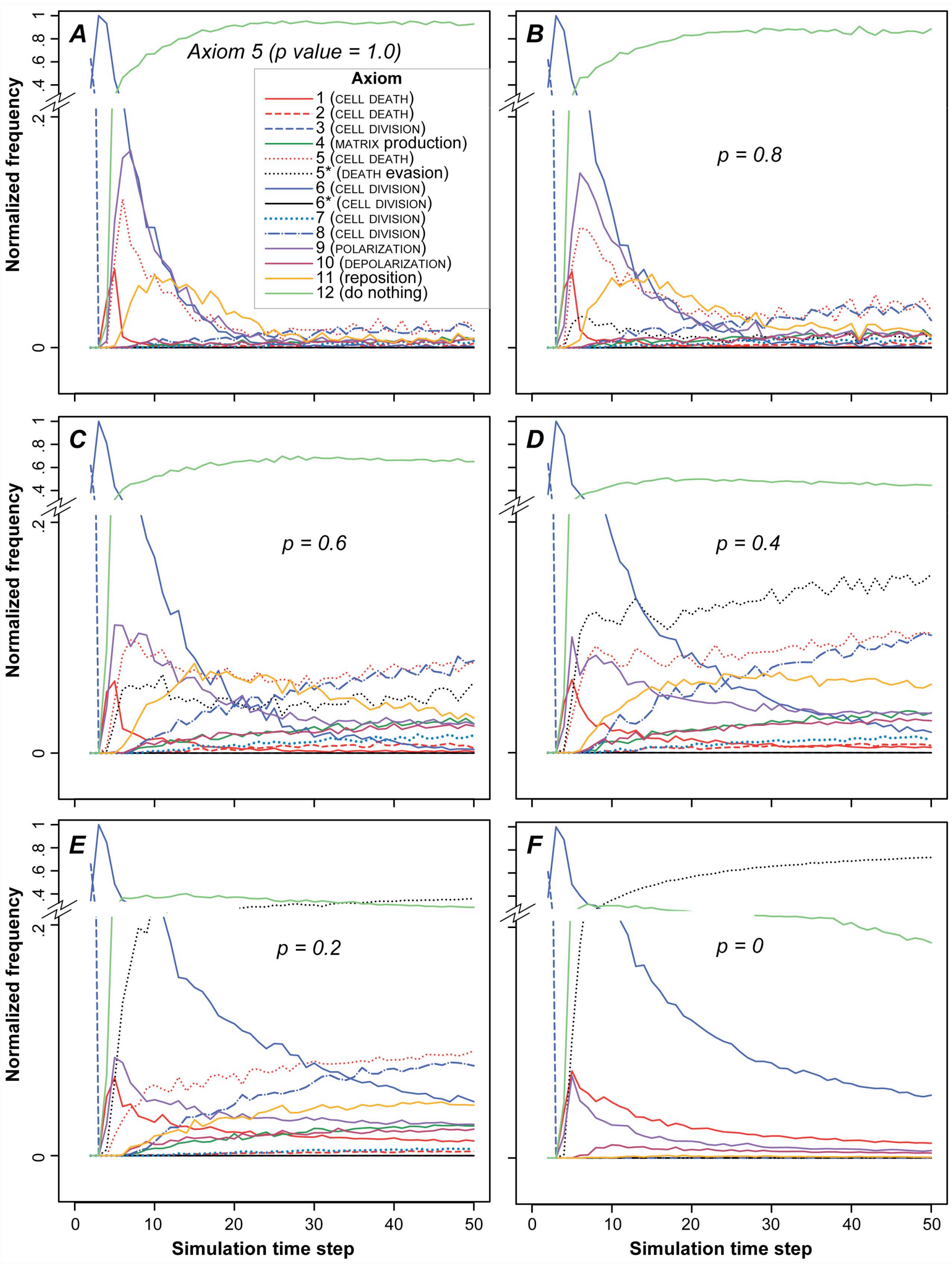

Figure S1. ISEA CELL axiom use patterns following Axiom 5 dysregulation. Axiom 5 dictates ANOIKIS (a form of CELL DEATH) when the CELL in its neighborhood has at least two CELLS and LUMINAL SPACE but no MATRIX. In simulation dysregulating Axiom 5, CELLS evaded ANOIKIS (i.e., do nothing) with a parameter-controlled probability, *p*, when Axiom 5's precondition was met. The use frequency was normalized so that individual frequency values sum to 1. The measurements represent mean values of 100 Monte Carlo outcomes.

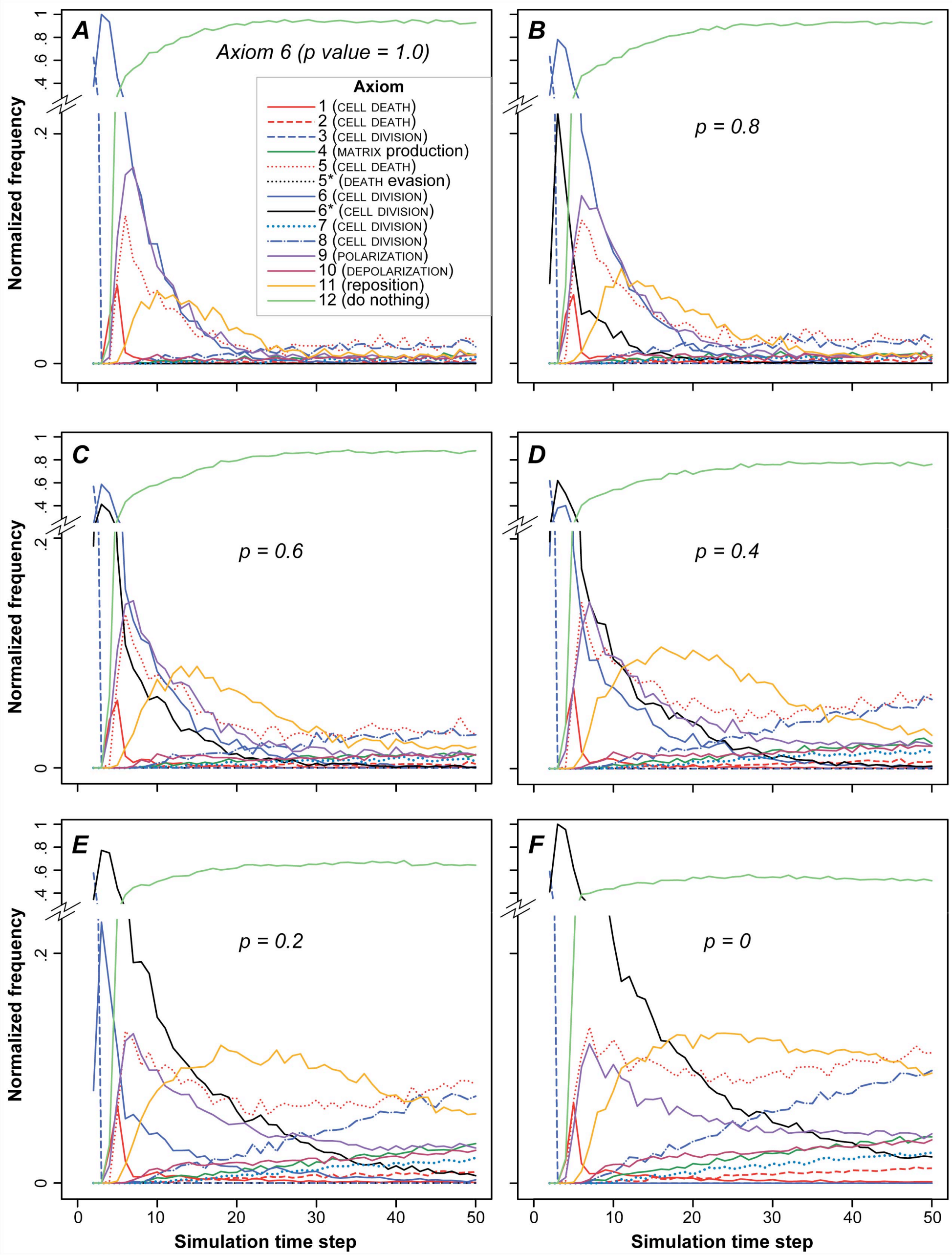

Figure S2. ISEA CELL axiom use patterns following Axiom 6 dysregulation. Axiom 6 dictates CELL DIVISION when the CELL has at least one CELL and MATRIX but no FREE SPACE in its neighborhood. The CELL copy is placed at an adjacent MATRIX position that maximizes its number of CELL neighbors. With a parameter-controlled probability, *p*, CELLS followed an alternate, dysregulated action (disoriented CELL DIVISION) when the Axiom 6 precondition was met. The CELL copy replaced a randomly selected MATRIX neighbor without regard for CELL neighbor number. The use frequency was normalized so that individual frequency values sum to 1. The measurements represent mean values of 100 Monte Carlo outcomes.

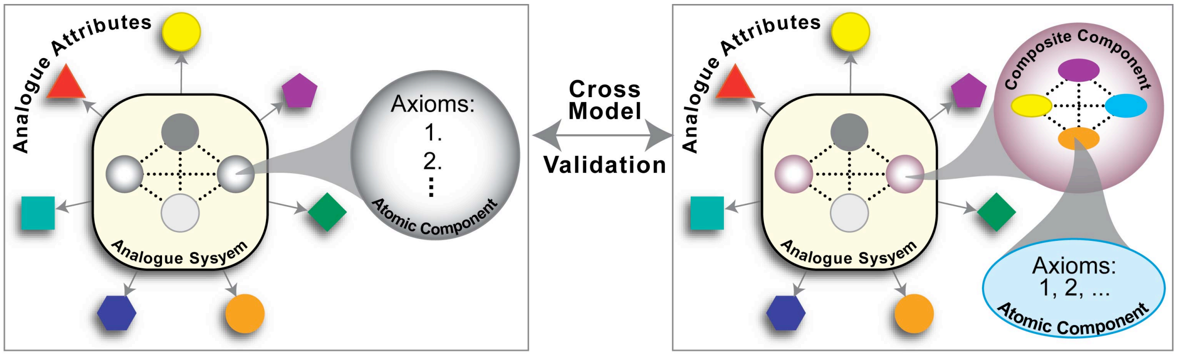

Figure S3. Model refinement and cross-model validation. CELLS are the main actors of the current AT II analogue. They are atomic components whose actions are governed by axioms. CELL actions and interactions cause phenotypic attributes. Because CELLS and other components are discrete objects, they can be replaced easily with new or revised objects. Replacements can be composite objects composed of other objects. Details at different scales can be incorporated into the analogue in like manner. When so doing, one must provide appropriate similarity metrics and demonstrate cross-model validation (original vs revised) to insure that the change does not alter analogue phenotype measurably.
